# Microbial Community Structure in Arctic Lake Sediments Reflect Variations in Holocene Climate Conditions

**DOI:** 10.3389/fmicb.2020.01520

**Published:** 2020-07-24

**Authors:** Tor Einar Møller, Willem G.M. van der Bilt, Desiree L. Roerdink, Steffen L. Jørgensen

**Affiliations:** ^1^Department of Earth Science, University of Bergen, Bergen, Norway; ^2^K.G. Jebsen Centre for Deep Sea Research, University of Bergen, Bergen, Norway; ^3^Bjerknes Centre for Climate Research, Bergen, Norway

**Keywords:** palaeoclimate, Greenland, microbial ecology, stratification, climate sensitivity, microbial stratification

## Abstract

The reconstruction of past climate variability using physical and geochemical parameters from lake sedimentary records is a well-established and widely used approach. These geological records are also known to contain large and active microbial communities, believed to be responsive to their surroundings at the time of deposition, and proceed to interact intimately with their physical and chemical environment for millennia after deposition. However, less is known about the potential legacy of past climate conditions on the contemporary microbial community structure. We analysed two Holocene-length (past 10 ka BP) sediment cores from the glacier-fed Ymer Lake, located in a highly climate-sensitive region on south-eastern Greenland. By combining physical proxies, solid as well as fluid geochemistry, and microbial population profiling in a comprehensive statistical framework, we show that the microbial community structure clusters according to established lithological units, and thus captures past environmental conditions and climatic transitions. Further, comparative analyses of the two sedimentary records indicates that the manifestation of regional climate depends on local settings such as water column depth, which ultimately constrains microbial variability in the deposited sediments. The strong coupling between physical and geochemical shifts in the lake and microbial variation highlights the potential of molecular microbiological data to strengthen and refine existing sedimentological classifications of past environmental conditions and transitions. Furthermore, this coupling implies that microbially controlled transformation and partitioning of geochemical species (e.g., manganese and sulphate) in Ymer lake today is still affected by climatic conditions that prevailed thousands of years back in time.

## 1. Introduction

Our ability to reconstruct past climate relies on the use of palaeoclimate proxies. These fingerprints of past climate conditions commonly comprise characteristics such as grain size measurements, mineral composition, organic matter content, geochemical signals tracking redox conditions, or biological signatures like plant spores and lipids, all of which are preserved in continuous geological archives like marine and lacustrine sediment sequences (e.g., Mayewski et al., 2004; Wanner et al., 2008; Sundqvist et al., 2014; Davies et al., 2015; Domaizon et al., 2017).

Alongside these conventional proxies, complex microbial communities are present in the sediments. Microbial cells that make up these communities are deposited on the sediment surface and buried over time (Starnawski et al., 2017), and the community composition is influenced by a number of environmental conditions that are ultimately controlled by the prevailing climate conditions at the time of deposition. For example, correlations are found between microbial community structures and stratigraphic variability in marine sediments that reflect changing redox conditions and organic matter availability (Inagaki and Nealson, 2006; Inagaki et al., 2015; Orsi et al., 2017; More et al., 2019). In addition, microbial abundance and activity in marine sediments are known to correlate with lithological characteristics resulting from changing depositional environments (Parkes et al., 2005; Picard and Ferdelman, 2011; Zinke et al., 2019). Similar connections have been observed in sediments from lacustrine settings, highlighting the important roles of catchment hydrology, lake water geochemistry and depositional conditions in controlling microbial populations (Vuillemin et al., 2016, 2018).

These findings tentatively suggest that microbial communities in marine and lacustrine sediment records may provide additional constraints to past climate reconstructions. However, at present we do not know how to translate their structure or composition into specific environmental conditions, thus disqualifying them as proxies sensu stricto. In addition, parts of the microbial population continue to be active and the relative and absolute abundances of individual taxa change over geological time (e.g., Parkes et al., 2005; Kirkpatrick et al., 2019; Zhao et al., 2019). This dynamic nature makes it even more challenging to link past climate conditions with a specific microbial population. Furthermore, the molecular signal typically used to infer the community structure could be distorted as it might comprise a combination of active, dormant and dead cells as well as extracellular DNA fragments (Pedersen et al., 2014; Domaizon et al., 2017; Ahmed et al., 2018; Ramírez et al., 2018; Ellegaard et al., 2020).

Here, we investigate links between palaeoclimate, environmental conditions and microbial community structure in two sediment cores from the seasonally ice-covered and glacier-fed Ymer lake on southeast Greenland. This study location is subject to rapid ongoing surface warming, caused by the amplified climate response of the Arctic (Serreze and Barry, 2011) and exposed to a number of climate-sensitive processes that are specific to Arctic lakes, such as ecological regime shifts, lake ice-climate feedbacks and changes in glacial erosion (Smol et al., 2005; Brown and Duguay, 2010; van der Bilt et al., 2016). Assuming a positive correlation between microbial response and climatic shifts, these major changes are likely to generate a microbial community structure in the sediment record that captures past environmental conditions. Our aim is to add data to the current body of literature in order to provide further constraints on the subject and investigate if the composition of buried microbial populations reflect climatic conditions at the time of deposition.

Our study builds on a previous palaeoclimate reconstruction based on sediment proxies by van der Bilt et al. (2018), and adds new data on pore water geochemistry, and microbial community structure. By applying multivariate statistics on these parameters, we show that the microbial population structure captures and reflects previously inferred shifts in palaeoenvironmental conditions at Ymer Lake. Using contrasting sedimentary records for the lower and upper basins of the lake, we show that while this reflection is captured irrespective of local settings, basin-specific features like water column depth and fluvial input nevertheless constrain the specific community in each sediment core.

## 2. Materials and Methods

### 2.1. Background and Regional Setting

**Figure 1:**
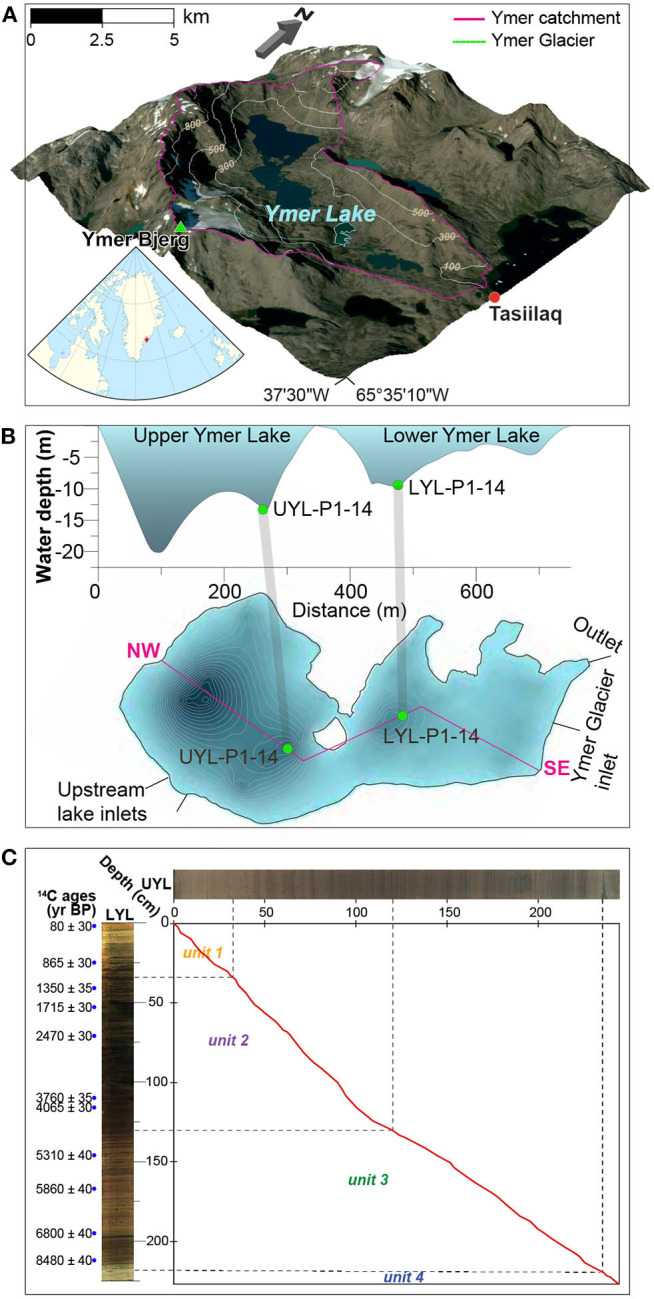
**(A)** Overview of the catchment area showing Ymer Lake (cyan), Ymer Glacier and the larger, unnamed up-stream lake. Embedded: Regional map showing Greenland and nearby land. Embedded regional map by Tentotwo, Wikimedia Commons CC BY-SA 3.0. **(B)** Bathymetric map of Ymer Lake. One piston core was gathered from each basin of Ymer Lake. 1B is modified from van der Bilt et al. (2018) with permission. **(C)** Correlation of sedimentation rates based on ^14^ C-dating of LYL (Betula leaves, *n* = 13) was performed using AnalySeries 2.0.8. (Paillard et al., 1996) based on linear interpolation of *n* = 59 tie points. Blue dots mark dated depths in LYL.

Our study site, the informally named Ymer Lake, is located on Ammassalik Island near the coast of South-East Greenland (Figure 1A) (65.37^o^N, 37.43^o^W). The lake measures 0.29 km^2^ and comprises two basins: the deep (max. 22 m) Upper Ymer Lake and the shallow (max. 10 m) Lower Ymer Lake (Figure 1B). The lake receives water from two main sources: the 0.9 km^2^ Ymer Glacier, perched in a cirque to the south with an inlet to the lower lake basin, and a much larger (3.5 km^2^) unnamed up-valley lake connected to the upper lake basin via a stream. More details about the catchment can be found in Supplementary Section 1.1 and in van der Bilt et al. (2018). We analysed one sediment core from each basin. A 248 cm long core from the Upper Ymer Lake (UYL-P1-14; abbreviated UYL) and a 228 cm long core from the Lower Ymer Lake (LYL-P1-14; abbreviated LYL). Henceforth, we will refer to the composite data of LYL and UYL as YL. Both cores were retrieved in July 2014 using a modified piston corer with an 11 cm diameter plastic coreliner. The cores have previously been used to reconstruct regional Holocene climate variability, and four lithological units have been identified (van der Bilt et al., 2018). In short: Unit 4 (10–9.5 cal. ka BP is characterised by deglaciation of the catchment area. Unit 3 (9.5–5 cal. ka BP), whose onset is marked by a Glacial Lake Outburst Flood (GLOF) deposit, is interpreted as the warm Holocene Optimum. Unit 2 (5.0–1.2 cal. ka BP) was deposited when the catchment became more prone to avalanches and flooding in response to Neoglacial climate deterioration. The transition to unit 1 (1.2–0 cal. ka BP) is marked by resumption of glacial activity (erosion) in the catchment as the still-present cirque glacier formed.

### 2.2. Sampling and Storage

**Figure 2:**
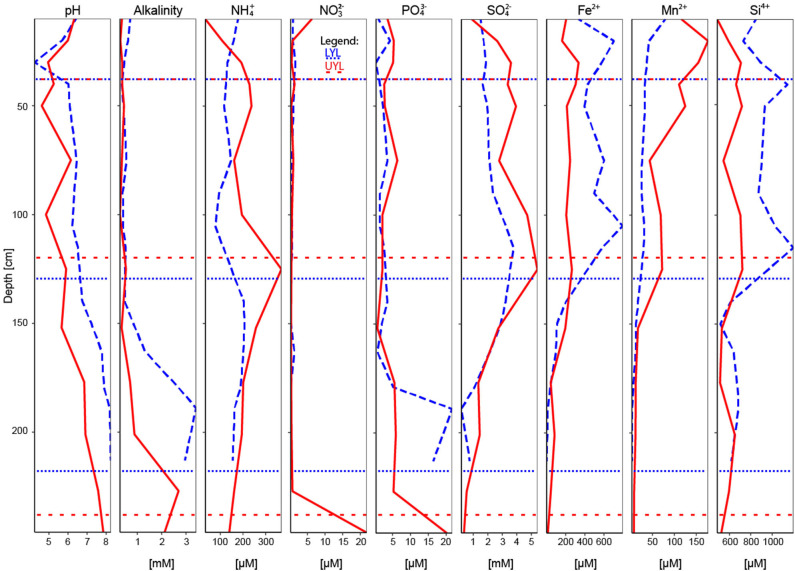
All pore water variables used in the study, including pH and alkalinity. Blue dashed line: LYL; red line: UYL. Horisontal dotted and spaced dotted lines refer to lithological separation into units for LYL and UYL, respectively.

The core was stored at 5°C for 2 months before it was split in halves and analysed by XRF scanning. After additional storage for 4 months, microbial and pore water sampling was performed. While we acknowledge that storage may have impacted our samples, the relatively high concentrations of dissolved Mn^2+^ and Fe^2+^ (Figure 2) throughout both cores suggest little or no reaction with oxygen during storage, which otherwise could drastically change both community structure and pore water composition. Nevertheless, we choose to remain very cautious about drawing inferences from presence to function for any specific microbial group in our data.

### 2.3. Microbial Sampling, DNA Extraction, Sequence Processing, and Quantification

The two cores were subsampled at regular depth intervals (17 from LYL and 14 from UYL, see Table 1 for details) using sterile cut-off syringes. Approximately 0.5 g of sediments was used to extract genomic DNA, applying the FastDNA® spin kit for soil from MPBIO according to manufacturer protocol. Two blank extractions were included to assess contamination. 16S rRNA gene amplicon libraries were prepared as follows: Extracted DNA was subjected to PCR amplification in duplicate using the primers Uni519F (5'-CAGCMGCCGCGGTAA-3') and 806R (5'-GACTACHVGGGTATCTAATCC-3'). Subsequent library preparation followed a previously described protocol (Jorgensen and Zhao, 2016). All libraries were pooled in a 1:1 ratio based on DNA concentration and sequenced using an Ion Torrent Personal Genome Machine (Life Technology, USA).

**Table 1:** Adjusted sampling horizons for microbial community composition and pore water extraction.

**Microbial horizon LYL [cm bsf]**	**Pore water horizon LYL [cm bsf]**	**Microbial horizon UYL [cm bsf]**	**Pore water horizon UYL [cm bsf]**
(5)		(5)	
10	8	10	8
20	15	20	15
30	24	30	25
40	36	40	35
50	46	50	45
75	69	75	70
90	86	100	95
105	103	125	120
115	122	152	149
140	144	177	174
150	157	201	199
163	167	227	224
179	182	246	242
189	192		
213	217		
(223)			

*To enable statistical comparison, some samples (in parentheses) were removed prior to constrained analyses. For each core, adjacent samples correspond in statistical analyses. bsf, below surface*.

After sequencing, all reads were filtered and clustered *denovo* at 97% similarity into Operational Taxonomic Units (OTUs) using the USEARCH and UPARSE algorithms (Edgar, 2010, 2013). Sequences were trimmed to 220 base pairs. Taxonomic classification of OTUs was performed using the program CREST with the SilvaMod reference database (Lanzen et al., 2012) built upon Silva SSURef nr release 106 (Pruesse et al., 2007), using the Lowest Common Ancestor algorithm. As contamination control, two blank samples were included in the pipeline, one for each core. 1) If an OTU in the sample data was represented by 20 sequences or less while being present in the blank, or 2) if the number of sequences in the sample data was less than one order of magnitude higher than the number of sequences in the blank, the OTU was discarded. This approach roughly follows previously published contamination control (Lee et al., 2015). Singletons were then removed, and samples were subsampled (rarefied) to 15058 reads, the lowest read count in any one sample. For further details regarding preparation of the 16S rRNA gene amplicon library, sequence processing, and taxonomic classification, we refer the reader to Supplementary Section 1.2.

### 2.4. Physical and Geochemical Analyses

**Figure 3:**
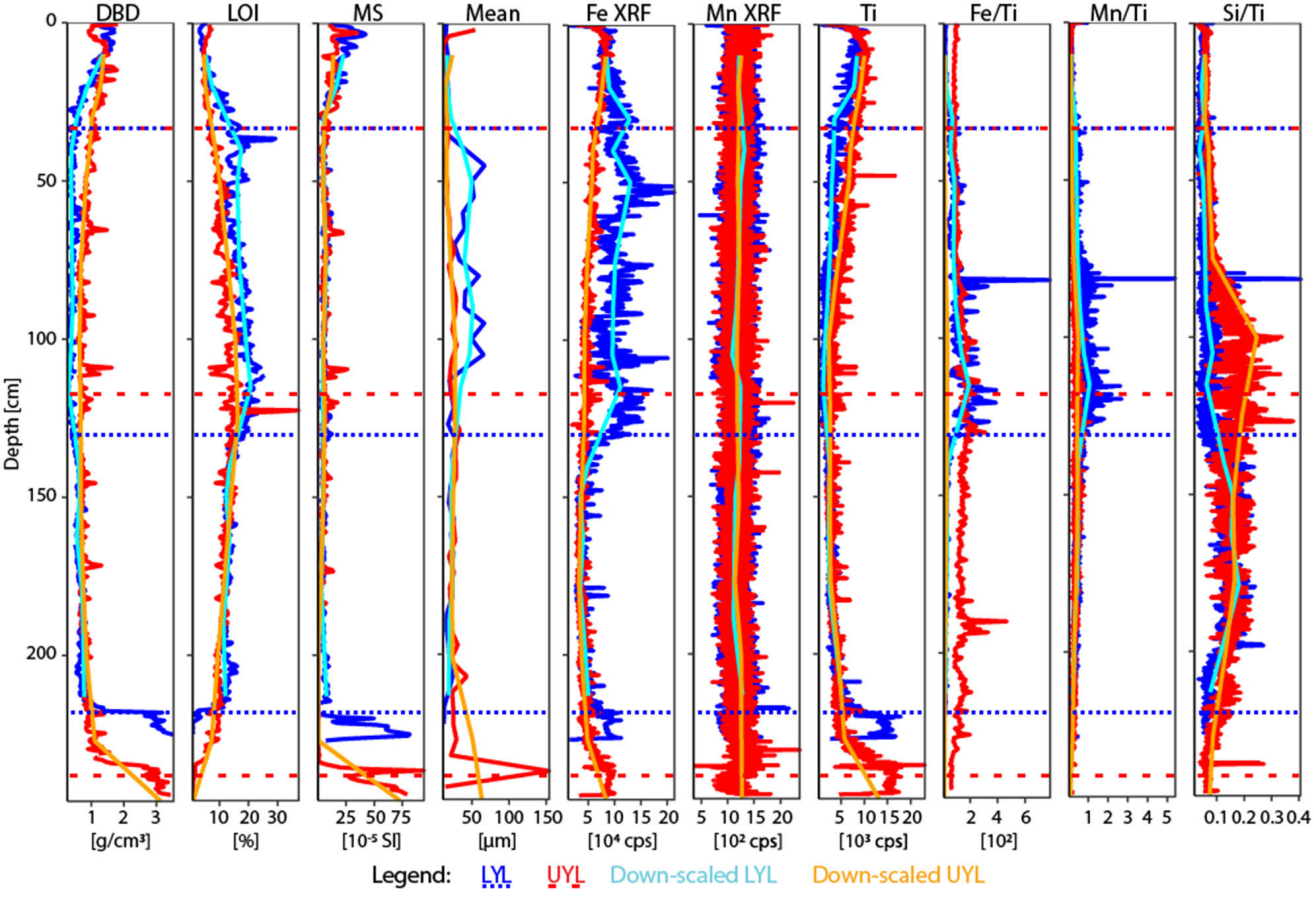
Physical and XRF proxy variables used in the study, plus additional redox-sensitive ratios Mn/Ti and Si/Ti. Mean is mean grain size. Blue line: LYL, high resolution; red line: UYL, high resolution; cyan line: LYL, upscaled resolution; orange line: UYL, upscaled resolution. Horisontal dotted and dashed lines refer to lithological separation into units for LYL and UYL, respectively. For XRF data, upscaling was performed using a running average of *n* = 50 for neighbours on either side of the center point. For DBD, LOI and MS, *n* = 4 and for mean grain size, *n* = 2.

The acquisition of down-core physical proxy variables and XRF elemental profiles is described in-depth by van der Bilt et al. (2018). Pore water was collected from regular depth intervals (see Table 1) using 0.2 μm Rhizon filters. A total of 15 samples from LYL and 13 from UYL were extracted and each split into four aliquots. One aliquot was analysed for pH using a mobile Metrohm 826 pH meter and alkalinity using a Metrohm 888 Titrando automatic titrator. A second aliquot was used for the measurement of nutrients (${\text{NH}}_{4}^{+}$, ${\text{NO}}_{3}^{2-}$, ${\text{PO}}_{4}^{3-}$) by photometric methods using a 4-channels Continuous Flow Analyzer (Seal Analytical Quaatro), and a third aliquot was analysed for anions (${\text{SO}}_{4}^{2-}$, Cl^−^) using an ion chromatograph (Metrohm). The fourth aliquot was acidified to 2% HNO_3_ and analysed for cations a Thermo Scientific iCAP 7600 inductively-coupled plasma optical emission spectrometer (ICP-OES) with Scandium as internal standard. Precision was better than 2% for all major elements reported in this study. From here on, the physical and geochemical variables are collectively referred to as context data. All context variables, in both high and low resolution, can be seen in Figures 2, 3, and a complete list of variables is provided in section 2.6.1.

### 2.5. Age-Depth Correlation

LYL was previously dated based on macrofossil (Betula leaves; *n* = 13) ^14^C ages as reported by van der Bilt et al. (2018). This chronology was used to produce an age-depth correlation with UYL and to align lithological units between the two cores. The correlation was established using version 2.0.8 of the AnalySeries software package (Paillard et al., 1996), and tie-points (*n* = 59) were selected by linearly interpolating Magnetic Susceptibility (MS) and Loss-on-Ignition (LOI) measurements (see section 2.6.1), as well as XRF-based Mn, Ti, and Ca counts.

### 2.6. Ordination and Clustering

To compare the microbial community structure's ability to capture palaeoclimate transitions to that of physical or geochemical proxy data and pore water composition, we performed a number of ordination and clustering analyses.

#### 2.6.1. Variable Selection and Sample Coercion

In line with the work of van der Bilt et al. (2018), we selected mean grain size, LOI, iron/titanium ratio (Fe/Ti), Dry Bulk Density (DBD), and MS as parameters to replicate the established separation into lithological units. We used conservative Ti counts to track minerogenic input (Bakke et al., 2009). Additionally, we included XRF-counts of iron (Fe-XRF) and manganese (Mn-XRF), as these may capture shifts in stratification brought about by, for example, changes in seasonal ice cover (Cuven et al., 2010). Finally, we included the XRF ratios Mn/Ti and Si/Ti, which may signify changes in redox-state (Davies et al., 2015).

To evaluate the ability of pore water to corroborate these proxy variables in detecting past climate transitions and constrain microbial response to past climate variability, we selected species and variables identified as biologically relevant and/or sensitive to microbially-mediated diagenesis. These include ${\text{NH}}_{4}^{+}$, ${\text{NO}}_{3}^{2-}$, ${\text{PO}}_{4}^{3-}$, ${\text{SO}}_{4}^{2-}$, and dissolved iron (Fe^2+^), manganese (Mn^2+^), and silicon (Si^4+^), in addition to pH and alkalinity (Berner et al., 1970; Froelich et al., 1979; Zeng et al., 2009; Glombitza et al., 2013).

Microbial data, i.e., all taxonomically assigned OTUs, were grouped on phylum, class, order and OTU levels. On all taxonomic levels, unassigned sequences were binned and labelled “No hits.” We then used the resulting taxonomic compositional distribution to investigate the microbial community's ability to capture/reflect palaeoclimate transitions. Multivariate analysis was performed using Principal Component Analysis (PCA). PCA was selected over non-Metric MultiDimensional Scaling (nMDS) to enable addition and interpretion of context data as response variables, see section 2.6.2 below for details.

To allow statistical comparison between the different data types, we coerced all data to the number of pore water samples that were sampled at the lowest resolution (Table 1). Physical and solid geochemical parameters were recorded at higher resolution than other data and were therefore averaged over a set of nearest neighbours aligned to respective microbial sampling horizons. From now on, samples are referred to by their core, plus sample depth in centimetres. For one horizon (UYL 246), we could not obtain a complete set of neighbours, so downstream analysis was performed on the incomplete set. See Supplementary Section 1.3 for details, including a complete list of neighbour omissions.

#### 2.6.2. Statistical Methods

To resolve the closed composition of the microbial data and remove any forced correlations, we log-transformed with zero-replacement all columns on all levels (Aitchison, 1982; Martín-Fernández et al., 2003). The context data was subjected to column-wise Hellinger transformation to ameliorate variability (Legendre and Gallagher, 2001). PCA was performed on phylum, class, and order levels. To determine the subset of context variables that best explain the variance expressed by the microbial community, henceforth referred to as the minimum adequate model, we used the iterative ordistep function (both forward and backward selection, otherwise default settings) in the R package vegan (Oksanen et al., 2018). In order to ensure the selection of a robust minimum adequate model, we ran the function 100 times for each core and taxonomic level. Selected models were subsequently controlled for variance inflation using the vif.cca function in vegan. We then performed Redundancy Analysis (RDA, a constrained version of PCA; van den Wollenberg, 1977) on the microbial data using the selected minimum adequate model variables as constraints to determine their explanatory power. Hierarchical Cluster Analysis with Euclidean distance and the square root of Ward's agglomeration criterion was used to assess sample clustering based on the two first resultant eigenvectors. All statistical analyses were performed in the R statistical programming environment (R Core Team, 2018). Analysis of similarities between groups of samples was performed using the anosim function, with 9999 permutations, in the R package vegan (Oksanen et al., 2018). In order to quantify the compliance between unconstrained (PCA) and constrained (RDA) analyses, we correlated their primary and secondary eigenvectors using Spearman's rho (ρ). In addition, we correlated the variance in the unconstrained microbial community structure with variance in selected geochemical variables to quantify their relationship, as previously done by Jorgensen et al. (2012).

## 3. Results

### 3.1. DNA Sequencing Analysis

After pre-processing, 2932 OTUs (97% similarity) remained in the dataset. We identified 2092 OTUs in LYL and 2495 in UYL, of which 1655 were present in both cores. OTUs were binned into 54 phyla, 97 classes, and 192 orders. Complete OTU tables for the aforementioned taxonomic levels, including blanks and discarded samples, are available in the supplement, as is a plot showing the distribution of the classes surpassing 1% relative abundance (Figure S1). Bacteria constituted 91.7% of all reads, Archaea 6.9%, Eukaryota 0.1%, and 1.3% could not be assigned to any domain. Proteobacteria was the most abundant bacterial phylum (24.7%), followed by Planctomycetes (11.2%), Chloroflexi (10.6%), Aminicenantes, previously Candidate Division OP8 (8.0%) and Atribacteria, previously Candidate Division OP9 (6.9%), respectively. The most abundant bacterial classes were Deltaproteobacteria (17.6%), Phycisphaerae (8.5%), and Chloroflexi Subdivision 6 (6.3%). Crenarchaeota was the most abundant archaeal phylum (5.6%), and the Bathyarchaeota, previously Miscellaneous Crenarchaeotic Group (5.6%) was the most abundant archaeal class. 17 phyla, 15 classes and 13 orders exceeded 1% relative abundance. We could assign 98.7% of all reads on phylum level, 76.7% on class level, and 53.6% on order level.

### 3.2. Physical and Geochemical Analyses

Dry Bulk Density (DBD) profiles are highly similar between UYL and LYL in units 4 (high values; ca. 10–9.5 cal. ka BP) and 3 (abrupt drop to low and stable levels; ca. 9.5–5.0 cal. ka BP), as seen in Figure 3. In unit 2 (ca. 5.0–1.2 cal. ka BP) and 1 (ca. 1.2–0 cal. ka BP), DBD values in LYL are significantly lower than in UYL, and the difference is highest around the onset of glacier growth in the catchment area (i.e., transition from unit 2 to unit 1). The inverse of DBD is true for Loss-On-Ignition (LOI) (Figure 3): Minima occur in unit 4 and 1, and a maximum around the transition from unit 2 to unit 3. LOI is approximately 6% higher in LYL than in UYL. Magnetic Susceptibility (MS) levels remain similar for both cores throughout, including concurring increases in unit 1 and 4. Mean grain size doubles on average, from 19.5 ± 4.1 μm in to 39.6 ± 16.2 μm, for unit 2 of LYL. This behaviour is not seen in UYL, where the measurements are stable (22.3 ± 6.1 μm) except for two extreme observations in unit 1 and 4, respectively.

Mn-XRF remains constant and largely invariant throughout both cores (Figure 3). Fe-XRF, on the other hand, displays a pattern similar to that observed for LOI; Concurring profiles throughout unit 4 and 3, before counts and variability drastically increase in LYL around the transition between unit 3 and 2, whereas UYL retains its trajectory towards slightly elevated levels in unit 1. Levels for the immobile minerogenic indicator Ti diverge in unit 2 and partly in unit 1, with a marked drop-off in LYL.

Total NO (nitrate plus nitrite) concentrations peak in both the top and bottom of UYL, reaching a maximum of 21.8 μM in unit 4. Concentrations in LYL remain comparatively low, never surpassing 1.7 μM. ${\text{SO}}_{4}^{2-}$ levels reach maxima of 3.77 mM at 122 cm in LYL and 5.41 mM at 120 cm in UYL, roughly corresponding to the transition between unit 2 and 3 in both cores (Figure 2). Further down-core, a steep decline in concentration coincides with an increase in alkalinity throughout unit 3 and 4 (Figure 2). ${\text{PO}}_{4}^{3-}$ exhibits a similar pattern, fluctuating between 1 and 6 μM throughout units 1 to 3 before peaking at around 20 μM in unit 4 in both cores. Mn^2+^ and Fe^2+^ concentrations decline to less than 1 μM in unit 4 in both cores, but Mn^2+^ concentration is initially much higher in UYL (max 177 μM) than in LYL (max 79.4 μM), and vice versa for Fe^2+^ (max 795 μM in LYL vs. 334 μM in UYL). Non-zero concentrations of dissolved Mn^2+^ and Fe^2+^ at the sediment/water interface in both core indicate that bottom waters in both LYL and UYL are anoxic.

### 3.3. Age-Depth Correlation

Linear interpolation (section 2.5), correlating UYL with already dated LYL, indicates that sedimentation rates have remained nearly identical in UYL and LYL during the last millennium, placing the boundary between unit 1 and 2 at around 35 cm sediment depth in both cores (Figure 1C). Sedimentation rate was higher in LYL than in UYL between 1.0 and 4.0 cal. ka BP. By this token, the transition boundary between unit 1 and 2 in UYL is set at 120 cm (130 cm in LYL). Between approx. 4–9 cal. ka BP the ratio of sedimentation in UYL and LYL lies stably around 3:2. Consequently, the transition between unit 3 and 4 in UYL is set at 238 cm (218 cm in LYL), implying that there are no unit 4 samples in LYL, for which 213 cm is the deepest horizon.

### 3.4. Unconstrained Ordination

#### 3.4.1. Physical and Geochemical Variables

**Figure 4:**
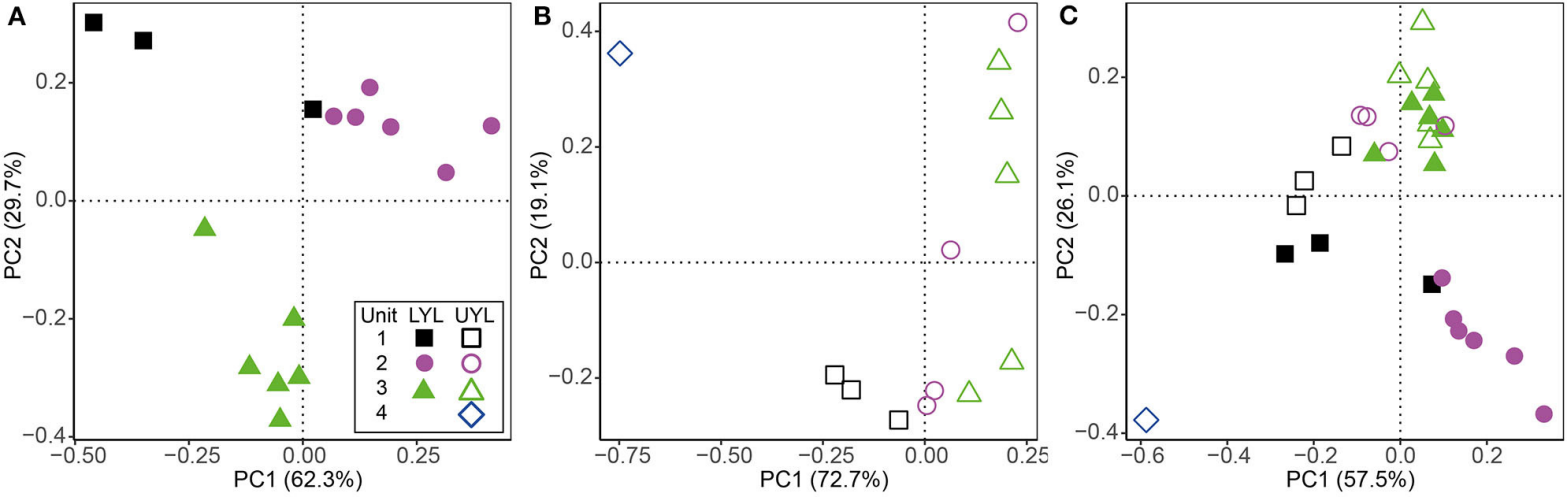
Ordination plot for first and second axes of variance from PCA of physical and XRF variables. **(A)** LYL (filled shapes), **(B)** UYL (hollow shapes), and **(C)** YL. Unit 1: black squares; unit 2: purple circles; unit 3: green triangles; unit 4: blue diamonds. Axis labels denote the percentage of total variance explained by the respective principal components (PCs).

Results from ordination and cluster analysis using the proxy variables DBD, LOI, MS, mean grain size, Fe-XRF, Mn-XRF, Ti, Fe/Ti, Mn/Ti, and Si/Ti on LYL, UYL, and YL are shown in Figure 4. For LYL (Figure 4A), we see distinct groups for unit 2 and unit 3, however, these are not fully separated from unit 1 samples in the associated cluster dendrogram (Figure S2A). The relative within-unit variability was higher in UYL (Figure 4B), resulting in dendrogram clusters (Figure S2B) not capturing the sediment gradient as clear-cut as for LYL. UYL 246 stands out as an extreme observation, indicating a very different sedimentological regime in unit 4. The composite of both cores, YL (Figure 4C and Figure S2C), shows that whereas unit 3 is very similar between the two cores, the remaining samples do not overlap in their clustering, with the highest discrepancy in unit 2 and 4.

Along the main axis of variance, the pore water variables (${\text{PO}}_{4}^{3-}$, ${\text{SO}}_{4}^{2-}$, total NO, ${\text{NH}}_{4}^{+}$, Mn^2+^, Fe^2+^, Si^4+^, pH and alkalinity) in LYL display a gradient from negative values in unit 1 and 2, toward increasingly positive values with increasing sample depth (Figure S3A). Samples from unit 1 and 2 cluster together (Figure S2D), but unit 1 displays higher dispersion among samples. In UYL (Figure S3B), samples from unit 1, 2 and 3 are placed increasingly positively along the second axis of variance, but with largest dispersion in unit 3. In both UYL and YL, UYL 246 again stands out as an extreme observation (Figures S3B,C), which is likely associated with the observed NO spike (Figure 2). In YL (Figure S3C), all unit 1 and 2 samples cluster together with little dispersion (Figure S2F), whereas samples from unit 3 spread out more, this time more distinctly along the main axis of variance instead of the second, as is the case for the individual cores (Figures S3A,B).

#### 3.4.2. Microbial Community Structure

**Figure 5:**
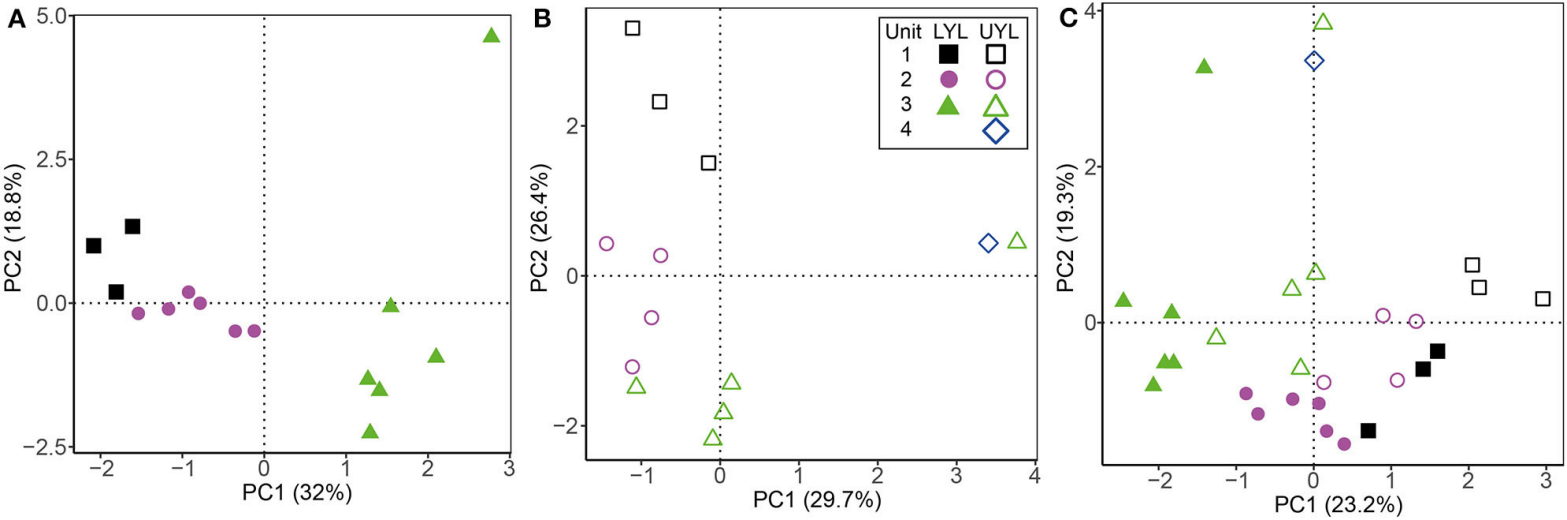
Ordination plot for first and second axes of variance from PCA of the microbial community composition in LYL on class level. **(A)** LYL (filled shapes), **(B)** UYL (hollow shapes), and **(C)** YL. Unit 1: black squares; unit 2: purple circles; unit 3: green triangles; unit 4: blue diamonds. Axis labels denote percentage of total variance explained by the respective principal components (PCs).

Based on visual inspection of initial ordination results and fraction of unassigned OTUs, the class level was found to carry the optimal trade-off between precision and taxonomic coverage, and was selected for downstream analysis. Resulting scatter plots from PCA of all microbial classes identified in LYL (Figure 5A) shows a distinct clustering of samples into lithological units. The main divide appears to be between unit 1 and 2 on one hand, and 3 on the other (Figure S4A), placing them negatively and positively along the main axis of variance, respectively. Notably, LYL 213 positions in the extreme top-right corner despite belonging to unit 3, albeit only five cm above unit 4, indicating a highly distinct microbial community compared to all other samples. In UYL (Figure 5B), UYL 227 and UYL 246 now cluster together, contrary to the physical variables (Figure 4), and appear to make up the majority of spread along the main axis of variance. The two samples from the deepest horizons are grouped closer to samples from unit 1 than from unit 3 in the cluster dendrogram (Figure S4B). The scatter plot from PCA on YL (Figure 5C) shows a systematic offset between the two cores. The microbial community in unit 4 plus UYL 227 from unit 3 is highly similar, and highly dissimilar to other communities (Figure S4C), likely due to their high abundance of Nitrospirae: 49.0% of reads in LYL 213, 13.7% in UYL 227, and 14.9% in UYL 246.

### 3.5. Constrained Ordination of Microbial Community Structure

RDA is a version of PCA where the primary axes of variance are defined as constraints imposed by the user. In this study, we use RDA to explore the effect of minerogenic input and redox-sensitive variables on the microbial community structure. The minimum adequate model is defined as the optimum set of constraints for explaining the variance expressed by a given dataset (see also section 2.6.2). The selected variables are then imposed as constraints, in our case on the microbial community structure, during RDA.

**Figure 6:**
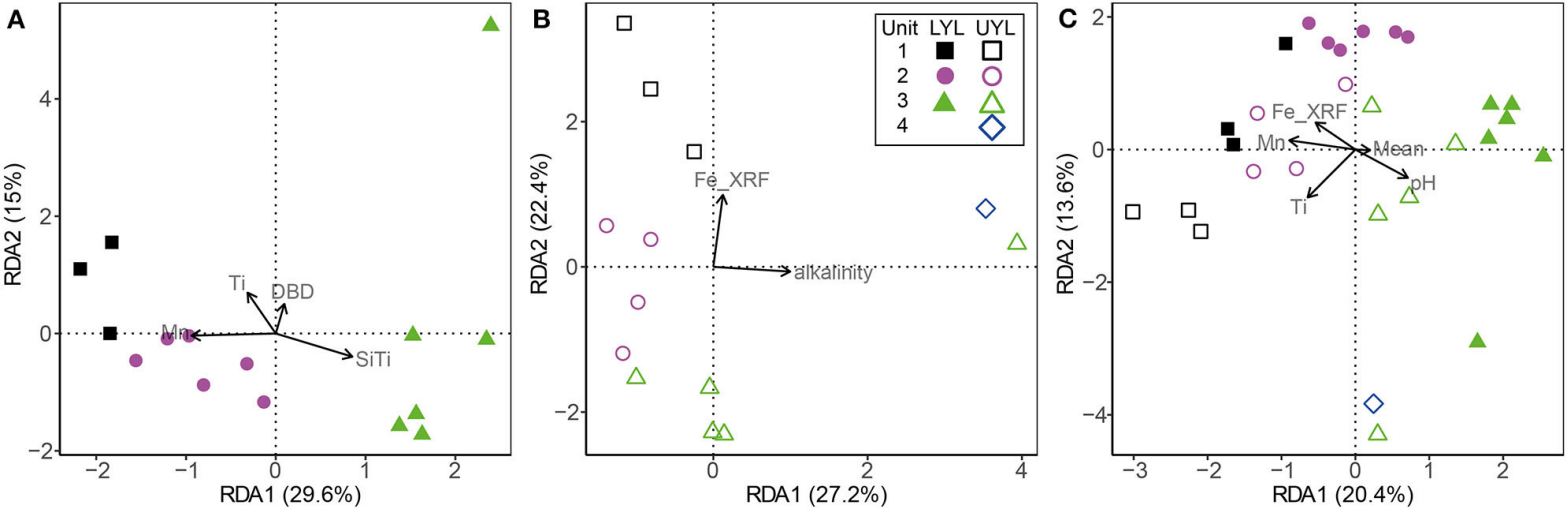
Ordination plot for first and second axes of variance from RDA of the microbial community composition in LYL on class level. **(A)** LYL (filled shapes), **(B)** UYL (hollow shapes), and **(C)** YL. Constraints were selected to constitute a minimum adequate model best explaining the variance in the microbial community structure data. Unit 1: black squares; unit 2: purple circles; unit 3: green triangles; unit 4: blue diamonds. Axis labels denote percentage of total variance explained by the respective principal components (PCs).

For LYL (Figure 6A), the variance inflation factor for LOI and DBD as calculated by the vif.cca function in the R package vegan exceeded 10 (Oksanen et al., 2018). Variance inflation is a measure of overlap of variance explained by two or more variables, and so a high value is indicative of redundancy. LOI had the highest factor and was subsequently removed as advised in the function documentation. The two main constraints are first Mn^2+^, then Ti, and these two are weakly correlated. Mn^2+^ correlates negatively with unit 3 samples, reflecting its low concentration in the pore water profile (Figure 2). Ti correlates positively with samples associated with glacial activity in the catchment. Alkalinity and Fe-XRF were the two main constraints identified for UYL (Figure 6B). These are entirely uncorrelated. UYL 125 is clustered with unit 2 in the dendrogram (Figure S4E), reflecting the transition from unit 3 to unit 2 in the palaeoclimate record. UYL 227 again groups distinctly with UYL 246 (Figure S4E) and is strongly positively correlated with alkalinity. Fe-XRF seems to account for most of the structuring of unit 1–3. Additionally, Ti and Fe-XRF correlate strongly (ρ = 0.90, *p* < 2.2·10^−16^), and the high variance inflation factor between the two show that they account for the same variance in the dataset. However, Ti explains slightly less variance then Fe-XRF: 21.7%. A total of five constraints: Mn^2+^, LOI, Fe-XRF, Fe/Ti, and alkalinity, were selected for the YL minimum adequate model (Figure 6C). LOI is negatively correlated with unit 1 and 4 samples, but positively correlated with unit 2 in LYL, reflecting the higher average LOI content in LYL compared to UYL (Figure 2). In line with down-core increase, alkalinity weakly correlates with unit 3 and 4 samples (Figure 2).

### 3.6. Analysis of Similarities

We perform analysis of variance (anosim) on class-level, transformed microbial data with Euclidean distance as appropriate (Aitchison, 1982; Gloor et al., 2017). The relative similarity between cores is highest in unit 3: *R* = 0.41 (*p* = 0.0051). The relative similarities in unit 1 and unit 2 are far lower, at *R* = 0.81 (*p* = 0.1) and *R* = 0.83 (*p* = 0.004), respectively. Unit 1 only consists of 3 samples in either core, hence 719 permutations exhausts all possible combinations for this comparison, and the associated significance level cannot fall below 0.1.

### 3.7. Correlations Between Variables and the Microbial Community

To quantify the relationship between the microbial community structure and its surrounding environment directly, we correlate eigenvectors from PCA and RDA with each other. The obtained correlation for primary eigenvectors (Figures S5A–C) are both above 0.9 (absolute value), and highly significant (*p* < 2.0·10^−6^). However, the correlation for UYL is heavily reliant on UYL 227 and UYL 246 for its significance (Figure S5B). For the secondary eigenvectors (Figures S5D–F), correlations all attain ρ > 0.79 (absolute value, *p* < 3.0·10^−4^). These correlations quantify the strong correspondence between the unconstrained and constrained ordinations and also verify that the selected set of context variables explain the main axes of variance in the microbial community.

Following Jorgensen et al. (2012), we also correlate primary eigenvectors from PCA on the microbial community with variables selected first by ordistep to directly quantify the effect of the latter on the former. Dissolved Mn^2+^, the main structuring variable for LYL (Figure S6A), correlates strongly (ρ = 0.897) and highly significantly (*p* = 5.29·10^−6^) with the corresponding primary PCA eigenvector. The analogous correlation between alkalinity and the primary PCA eigenvector of UYL (Figure S6B) is even stronger (ρ = 0.946, *p* = 9.83·10^−7^), but relies on UYL 227 and UYL 246 in order to be significant. Finally, there is a strong (ρ = 0.822, *p* = 8.41·10^−8^) correlation between the primary PCA eigenvector and dissolved Mn^2+^, the main structuring gradient for YL (Figure S6C).

## 4. Discussion

### 4.1. Clustering of Geochemical and Physical Variables in Ymer Lake

The Holocene sedimentary records presented here have previously been subdivided into four lithological units, each representing intervals characterised by distinct climatic conditions (van der Bilt et al. 2018; see also section 2.1). This was done using a multi-proxy approach combined with visual logging. Using statistical approaches (clustering and ordination) on a spatially up-scaled subset of these proxies, our analyses largely reproduce the subdivision into these lithological units, particularly in LYL (Figures 4A,B and Figures S4A,B). However, we also note that there are some pronounced deviations where one or several samples from one unit cluster together with samples from a different unit (Figure S2). While this, at least to some extent, could be explained by the use of up-scaled data collected along a gradient, it highlights the importance of visual and contextual guidance when lithological units are defined.

In addition to geochemical variables in the solid phase (scanning XRF), we also perform ordination and clustering based on the geochemical variation in the pore waters (Figures S2D–F, S3). These results show no clear separation between units and only unit 3 (ca. 9.5–5.0 cal. ka BP) in LYL is clearly defined. Geochemical depth profiles of pore water composition results from the sequential depletion of electron acceptors during organic carbon mineralisation (Froelich et al., 1979). The sequence in which the electron acceptors are depleted is dictated by Gibbs free energy and largely independent from climate conditions. In addition, pore water is mobile and partly controlled by diffusion rates. Hence, as the boundaries are expected to be less defined, ordination and clustering using pore water composition seems less likely to reproduce a clustering pattern into lithological units.

### 4.2. Microbial Variability and Relation to Depositional Conditions

The microbial community profile in both cores largely group according to the previously inferred four lithological units (Figures 5A,B and Figure S4), and even more so than the physical and XRF variables (Figures 4A,B and Figures S2A–C). This finding strongly suggests that the contemporary signal of the microbial community is still influenced by conditions prevalent during the time of deposition, even after thousands of years of burial. In that respect, our findings reflect those of e.g., Vuillemin et al. (2016, 2018), who also correlate distinct microbial communities with different sediment lithology. While this type of analysis shows that the combined influence from past climate are still imprinted in the community composition, it does not suggest which specific factors explain this linkage. In an attempt to resolve this question, we compiled our context variables into a single data table and compared their explanatory power. By doing so, we show that the variables explaining most of the microbial variance are potentially products of microbial activity; Mn^2+^ and alkalinity in LYL and UYL, respectively. It might seem contradictory that, on the one hand, community structure is linked to past depositional conditions, while the variance is best explained by pore water constituents largely independent from past climate on the other. However, it is important to note that not all variance is explained by Mn^2+^ and alkalinity (29.6% in LYL and 27.2% in UYL, respectively). Moreover, causality cannot be inferred from ordination analyses or correlations alone, hence the results need to be contextualised before qualified interpretations about the direction of forcing can be made. In this case, it seems reasonable to conclude that the community structure and its related activity causes the close linkage and not vice versa. Furthermore, it points toward a community that is at least partly active. This notion is supported by the very tight correlations between PCA eigenvectors and Mn^2+^ and alkalinity (Figures S5, S6). These correlations would be highly unlikely if the community variability inferred from the DNA fingerprint originated chiefly from dormant or dead cells rather than an active population.

The second best explanatory variables are XRF-counts of titanium (Ti) in LYL and iron (Fe) in UYL, respectively. We note that Fe has a very strong correlation with Ti (ρ = 0.90, *p* < 2.2·10^−16^) in UYL, suggesting that both capture the same process and that Ti also in UYL is a powerful explanatory variable (see section 3.5 for details). This tight linkage between microbial variability and Ti in both cores is further substantiated by the highly significant correlation when the composite dataset is analysed ρ = 0.66 with *p* < 0.0002 (Figure S7A). Ti is a variable that tracks minerogenic clastic input, a parameter that is closely linked to climate-sensitive processes like weathering, flooding and run-off (van der Bilt et al., 2015). As Ti is also considered redox insensitive and unaffected by microbial activity, it allows us to predict the direction of forcing with a high degree of confidence; variability in minerogenic input has a significant impact on the microbial community structure, directly or indirectly.

Our results suggest that the microbial communities in the sediments are metabolically active with a consequently tight link to pore water constituents associated with metabolic processes. We also show that major environmental (depositional) transitions are identified by co-occurring shifts in microbial populations. The latter retain a quantifiable link to variables proposed by van der Bilt et al. (2018) to be driven by changing climate, especially those linked to changes in the input of minerogenic material. While these results give strong hints to the direction of forcing, testing for any mechanistic coupling between depositional conditions and microbial community structure is beyond the scope of this study. However, by comparing sedimentary records from each of the two connected basins of Ymer Lake (Figure 1B), we outline how local, site-specific, manifestations of regional climate forcing may constrain deposited microbial communities.

### 4.3. Local Settings Constrain Climatic Influence on Microbial Populations

The two investigated sites are located only 200 meters apart and thus experienced the same climatic conditions, yet our results demonstrate inter-basin variability in physical, geochemical and microbial parameters (Figures 4C, 5C). While sediment input to the lower and upper lake show little variation during deposition of unit 3 (Figures 3, 4C), corresponding to the Early-to-Mid Holocene (ca. 9.5–5.0 cal. ka BP), the depositional conditions in the two basins started to diverge abruptly around the regional transition to a cooler climate during the Late Holocene after ca. 5.0 cal. ka BP (Briner et al., 2016; Axford et al., 2017; van der Bilt et al., 2018). This is reflected in the values of several physical parameters (i.e., LOI, DBD, mean grain size, MS), and redox state as seen in Figures 3, 4C. We argue that the marked difference in water depth (Figure 1B) is likely to be responsible for part of the observed increased deviation. One of the assumed consequences would be a more reduced environment in the shallower and smaller lower lake. This notion is supported by the elevated levels of organic carbon content (LOI) and redox sensitive parameters (Fe/Ti and Mn/Ti) observed here as compared to the upper lake (Figure 3). We also note that the upper and lower lake system receive fluvial input from different sources, namely an upstream lake and Ymer glacier, respectively. While this in itself is likely to cause some of the variation, one could speculate that a colder climate may have kept the shallow (< 0.5 m; Figure 1B) connection between the two basins closed by ice for longer periods, highlighting the potential differences in fluvial input more clearly. Overall, this suggests that despite experiencing similar climatic forcings, site-specific factors like water depth greatly affect the physical and geochemical signature in the deposited sediments.

It is clear from our combined ordination analyses (Figures 5C, 6C) that the contemporary microbial communities also differ between the upper and lower basins, irrespective of identical regional climate forcings. We furthermore note that our unit-wise comparative analysis (anosim) show that the communities are most similar in unit 3 (*R* = 0.41, *p* = 0.0051) and least so in unit 2 (*R* = 0.83, *p* = 0.004), thereby following the trend in variability observed for the depositional conditions. The same local settings that we claim contributed to the observed differences in sediment composition are also highlighted by Rogozin et al. (2009) as drivers of variability in limnic microbiota. The mechanisms behind this control are multiple but one of the more important is the control on redox state, particularly in seasonally or permanently ice-covered lakes like our study site (Coolen et al., 2004; Rogozin et al., 2009; Bertilsson et al., 2013; Schütte et al., 2016). The strong correlation between the secondary axis of microbial variability and the redox-sensitive ratio Fe/Ti across all our samples (ρ = −0.73, *p* = 1.2·10^−4^, data not shown), make us suggest that redox conditions remains an important factor shaping the microbial community in the sediments, as also noted for marine environments (e.g., Orsi et al., 2017). Our data do not allow to test if the initial population settling and developing in the surface sediments have indeed been different through time at the two sites. However, as the seeding population continuously adapts to the prevailing redox condition in the lake at any given time (Coolen et al., 2004; Rogozin et al., 2009; Bertilsson et al., 2013; Schütte et al., 2016; Thomas and Ariztegui, 2019), we find it more plausible that an initial strong linkage is maintained over time rather than developed after burial through changes in community structure. We therefore suggest that the initial microbial community established in the surface sediments differed between the two sites. While they are very likely to evolve over time, they maintain their link to past depositional conditions.

When searching for other explanatory factors related to microbial variability we again wish to highlight the strong and significant positive correlation with minerogenic input (Ti), a link that is retained across the two basins and even strengthened during periods with large differences in depositional conditions (Figure S7). In this light, we note that Ti is also often used to track detrital input (e.g., Bakke et al., 2009) and its apparent anti-correlation to LOI in our samples supports such use. This, in combination with the strong correlations between the secondary microbial variability (PCA second eigenvector) and organic carbon (LOI) in the combined dataset lead us to suggest that variability in organic carbon input is another very strong influencing parameter on microbial variability, in line with previous studies of lake sediments (Nelson et al., 2007; Kallmeyer et al., 2015; Vuillemin et al., 2018).

In sum, our comparative analyses of the two sedimentary records show that variations in past depositional conditions, brought about by regional climate forcing, are captured in the contemporary microbial communities, and give hints to important underlying drivers such as lake redox conditions and organic carbon content. These drivers are directly influenced by regional climate; however, their manifestation is strongly dependent on local settings such as water depth and fluvial input. Hence, the direct link between climate conditions and microbial communities has been “filtered” through local settings.

## 5. Conclusions

By leveraging the potential of Principal Component and Redundancy Analyses (PCA and RDA), we show that the contemporary microbial community structure within the analysed cores capture past climatic conditions throughout the Holocene. In fact, it seems to record climatic shifts better than many physical and geochemical variables, highlighting how microbial profiles can add biological context and detail to an already established palaeoclimate reconstruction.

Our results suggest that the majority of the microbial community is active and strongly connected to factors directly linked to ongoing metabolic activity, but nonetheless retains a quantifiable linkage to depositional conditions associated with past climate history. It is important to note that this linkage to climate is concerned with the community's structural variability and not necessarily to the specific composition (i.e., the variance between taxa as opposed to the presence or absence of specific taxa in the community). We identify lake redox conditions and organic carbon content as potential underlying drivers of microbial community variability, in line with previous suggestions (Vuillemin et al., 2016, 2018). These drivers are constrained not only by regional climate forcing, but also by basin-specific settings such as water depth and fluvial input.

An important notion to the apparent linkage between microbial communities and past climate conditions is the coupling to ongoing geochemical cycling. As microbes, through their metabolic activity, regulate the partitioning of a number of important geochemical elements across the sediment-water interface (e.g., oxygen, nitrate, iron, manganese and sulphur), our observations imply that major changes in past climate are still influencing the water chemistry in the investigated lake today.

Although we are still far from understanding all the intricate feedback mechanisms between past climate and contemporary microbial activity, there is increasing evidence that they are intimately linked (e.g., Kallmeyer et al., 2015; Vuillemin et al., 2018; Zinke et al., 2019), suggesting that past climate is also likely to influence our current climate and the response to future change.

## Data Availability Statement

The datasets generated for this study can be found in the NCBI SRA under project number PRJNA607019 (16S rRNA sequence data). Physical and geochemical data are available on Pangaea as well as in the supplement.

## Author Contributions

TM, SJ, WB, and DR designed research. WB retrieved the core, did XRF scans and performed physical analyses of the cores. DR extracted and analysed pore water geochemistry. SJ extracted DNA and prepared samples for sequencing. TM performed statistical analyses and led the writing process. All authors were involved in the writing and editing process of the paper.

## Conflict of Interest

The authors declare that the research was conducted in the absence of any commercial or financial relationships that could be construed as a potential conflict of interest.
