# Supplementary material for: Microbial Community Structure in Arctic Lake Sediments Reflect Variations in Holocene Climate Conditions
Source: Front Microbiol. 2020 Jul 24;11:1520. doi: 10.3389/fmicb.2020.01520 (PMC7396534; doi:10.3389/fmicb.2020.01520)
Supplement: Supplementary file 1 [file Presentation_1.pdf]

Supplementary materials for

Microbial community structure in Arctic lake sediments reflect variations in Holocene climate conditions

Tor Einar Møller*, Willem G. M. van der Bilt,
Desiree Roerdink, Steffen L. Jørgensen*

* To whom correspondence should be addressed: tor.moller@uib.no or steffen.jorgensen@uib.no

1 Supplementary data

Raw 16S sequence data may be found on NCBI under accession number PRJNA607019. Physical, geochemical (XRF) and pore fluid (plus pH and alkalinity) data may be found separately as csv files. These data comprise mean grain size measurements, physical properties (magnetic susceptibility, loss-on-ignition, dry bulk density), X-Ray Fluorescence (XRF) data (Mn, Fe, Si, Ti), cations (Fe, Mn, Si) and various nutrients (NH4, NO3/NO2, PO4, SO4 as well as pH and alkalinity). All depth indications are given in cm, all concentrations in micromolar. Dry bulk density is given in g cm^{-3}, loss-on-ignition in %, magnetic susceptibility in 10^{-5} SI. XRF data is given in cps. Mean grain size in micrometres.

R scripts used to produce the results and figures presented in the paper may be accessed by request from T.E.M.

2 Supplementary methods

2.1 Regional setting

The lake‘s perimeter is mostly flat and vegetated, shielding both basins from mass-wasting processes, but the southern and northern limits of the catchment are flanked by alpine mountains (max. 916 m asl). Two bedrock formations underlie the catchment: 1) the basic igneous Ammassalik Intrusive Complex (AIC) that envelopes the lake shores, and 2) the metamorphosed sedimentary rocks of the Siportoq Supracrustal Association (SSA) that outcrops south of the lake Kolb (2014). At present, climate on Ammassalik Island is low Arctic maritime, with a measured mean air temperature of -4.5°C and 870 mm of average annual precipitation between 1895 and 2010 in nearby Tasillaq Cappelen et al. (2011). Beyond instrumental observations, previous paleoclimate investigations suggest that the retreating Greenland Ice Sheet vacated Ymer Lake around 10 cal. ka BP Lecavalier et al. (2014); van der Bilt et al. (2018).

2.2 Microbial DNA extraction and classification

2.2.1 Preparation of 16S rRNA gene amplicon library

Core subsampling was performed using a sterile cut-off 5ml syringe. DNA was extracted from approximately 0.5 gram of sample material by applying the FastPrep soil DNA isolation kit, and following the manufactures protocol. DNA was finally eluted in 100 μl PCR-grade double-distilled water (ddH_2O), and preserved at -80°C until further analysis. In order to assess potential contamination introduced from the extraction kit, two blank extractions were included using the same batch of chemical reagents as for the samples. All DNA extracts were PCR amplified in duplicates with the SSU rRNA gene specific primers 519f (5´CAGCMGCCGCGGTAA) and 805r (5´GACTACHVGGGTATCTAATCC) in order to generate a gene amplicon library for subsequent sequencing using the Ion Torrent PGM Personal Genome Machine (PGM) platform technology (Life Technologies). Each reaction (20 μl) contained 10 μl 2x HotStarTaq® master mixture (Qiagen), 0.2 μl of each primer (100 μM stock) and dH_2O. The PCR program was initiated with a hot start activation step for 15 minutes at 95 °C followed by 30 cycles of 95 °C for 30 seconds, 56 °C for 30 seconds and 72 °C for 30 seconds. The duplicate PCR products were pooled to minimize PCR drifting, and purified using QIAquick PCR purification kit (Qiagen). For the attachment of Multiplex Identifiers (MIDs) another seven cycles were run, where each reaction (25 μl) contained 12.5 μl 2x HotStarTaq® master mixture (Qiagen), 0.2 μl primer 806r-B-Key (100 μM stock) and 2 μl 519f MID primer (10 μM stock) according to the Ion Torrent protocol. The PCR amplicons were purified using AMPure XP bead Purification Kit (Agencourt), following manufactures protocol, before all samples were pooled (26 pM per sample).

2.2.2 Microbial abundance quantification

Down-stream 16S rRNA gene sequence analysis includes the following steps: Sequences where filtered and clustered into operational taxonomic units (OTUs) using the USEARCH and UPARSE algorithms (Edgar, 2010, 2013). Quality filtering and trimming to 220 bp was performed with the '-fastq_filter' command using options '-fastq_trunclen 220' and '-fastq_maxee 1'. Chimeric sequences were detected and removed with the '-uchime_ref' command using the Gold database as reference. De novo OTU clustering was performed at a cutoff of 97% nucleotide sequence similarity using the'-cluster_otus' command. Taxonomic classification of OTUs was performed using the program CREST with the SilvaMod reference database Lanzén et al. (2012) built upon Silva SSURef nr release 106 Pruesse et al. (2007), using the Lowest Common Ancestor algorithm. Two blanks, one for each core, were first visually inspected and found to contain negligible numbers of sequences for most detected OTUs. We then proceeded to remove any OTU present in more than 10% relative abundance in any blank, removing all significant contaminants. We then discarded all singleton OTUs. Despite the known shortcomings of ordinary subsampling (also ambiguously referred to as rarefying or rarefaction; McMurdie and Holmes (2014); Gloor et al. (2017)), we still decided to proceed doing so to correct for systematically higher read count in UYL compared to LYL, and an anomalous read count in LYL_163 (470 000 reads compared to a median of 23 000). Subsampling was performed with respect to the smallest library (15058 reads) using the rrarefy function in vegan (v.2.5.2, Oksanen et al. (2018)). OTUs were grouped on order, class, and phylum levels. On all taxonomic levels, unassigned sequences were binned and labelled "No hits".

2.3 Variable selection and sample coercion

The following horizons were left out from sample averaging (variables in parentheses) (neighbours on either side in parentheses):

- Physical data (MS, LOI, DBD) (4): UYL_246. 246 cm was deeper than deepest corresponding physical data horizon. Only 5 out of maximum 8 data points used for averaging.
- XRF data (Fe XRF, Mn XRF, Ti, Fe/Ti, Mn/Ti, Si/Ti) (50): UYL_246. 246 cm was deeper than corresponding composite horizon. Only 51 out of maximum 100 data points used for averaging.

3 Supplementary figures

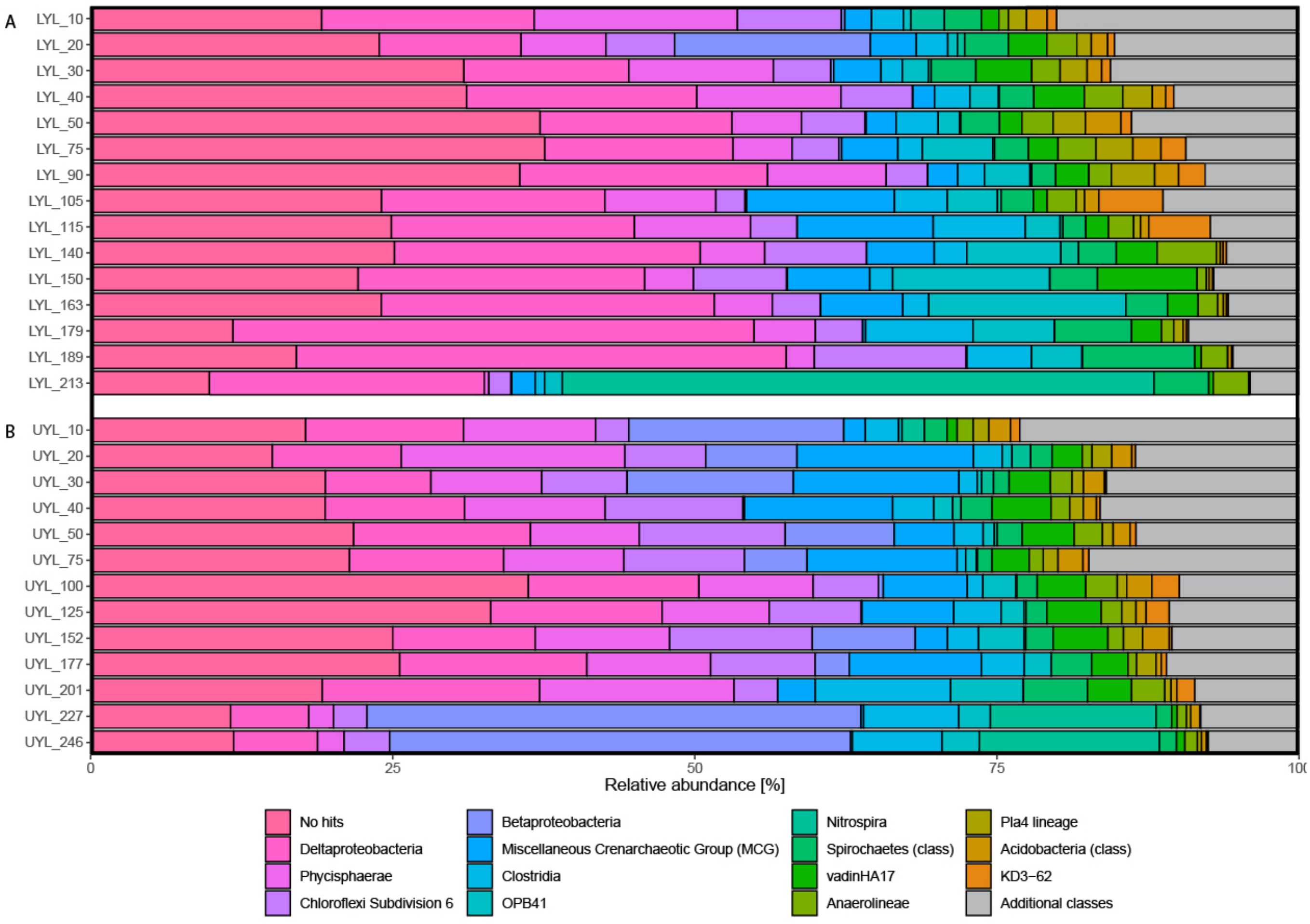

Figure 1: Normalised barplot showing the 14 classes exceeding 1% relative abundance in the data set, plus the binned class of unassigned reads (No hits) as well as additional, assigned classes. **(A)** LYL, **(B)** UYL.

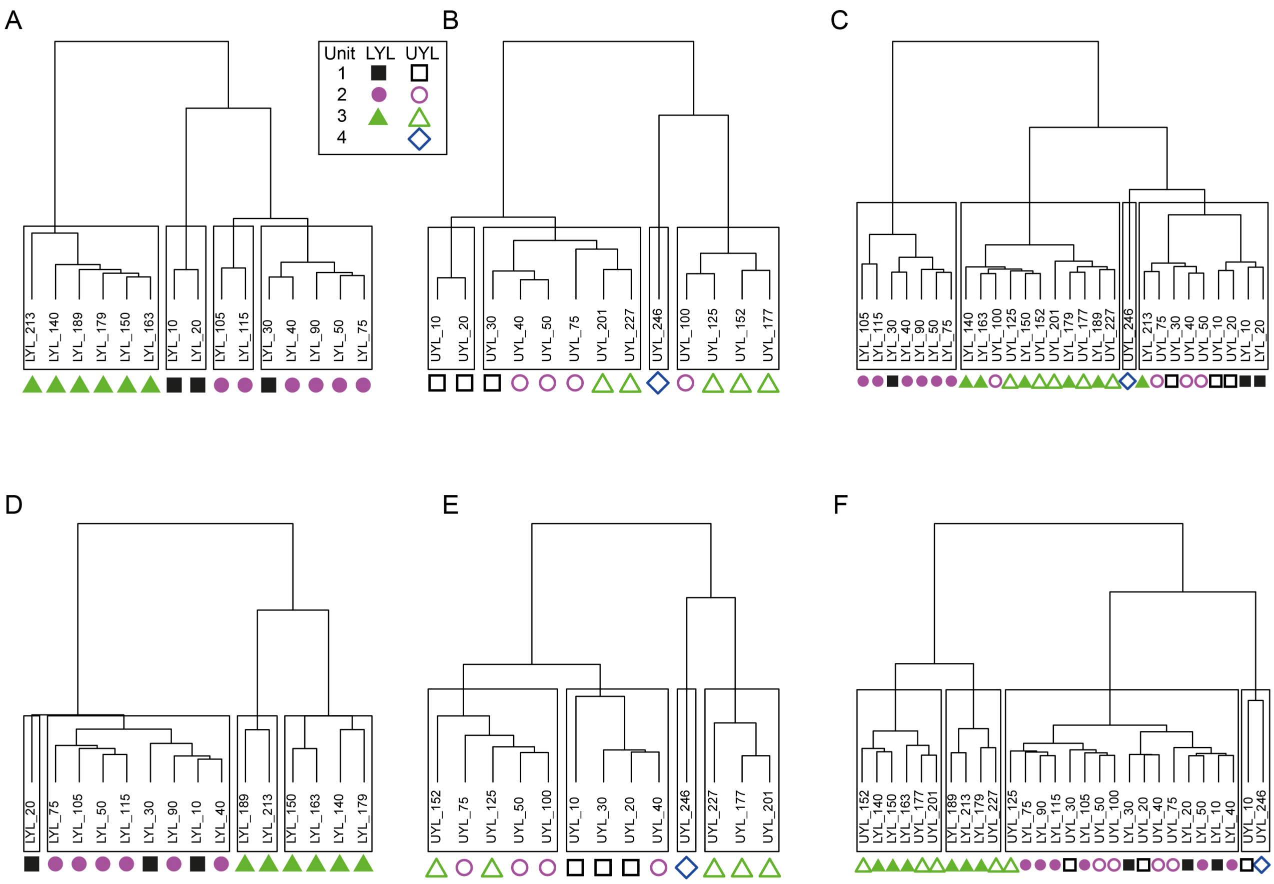

Figure 2: Cluster dendrograms for first and second axes of variance displayed in scatter plots of **(A-C)** PCA on physical and solid geochemical proxy variables, and **(D-F)** PCA of pore fluid variables, including pH and alkalinity. Unit 1: black squares; unit 2: purple circles; unit 3: green triangles; unit 4: blue diamonds.

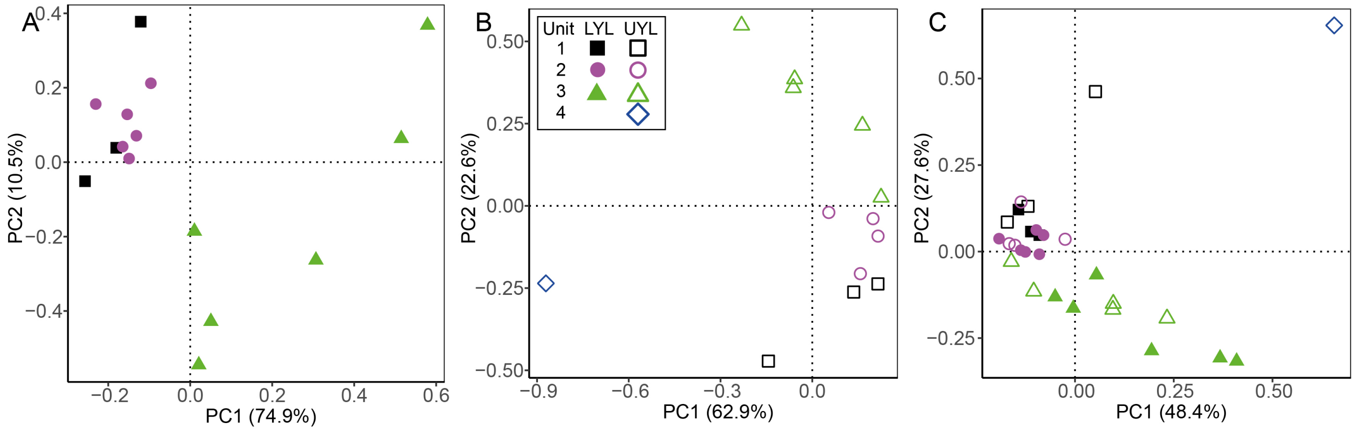

Figure 3: Ordination plot for first and second axes of variance from PCA of pore fluid variables, including pH and alkalinity. **(A)** LYL (filled shapes), **(B)** UYL (hollow shapes) **(C)** YL. Unit 1: black squares; unit 2: purple circles; unit 3: green triangles; unit 4: blue diamonds. Axis labels denote percentage of total variance explained by the respective principal components (PCs).

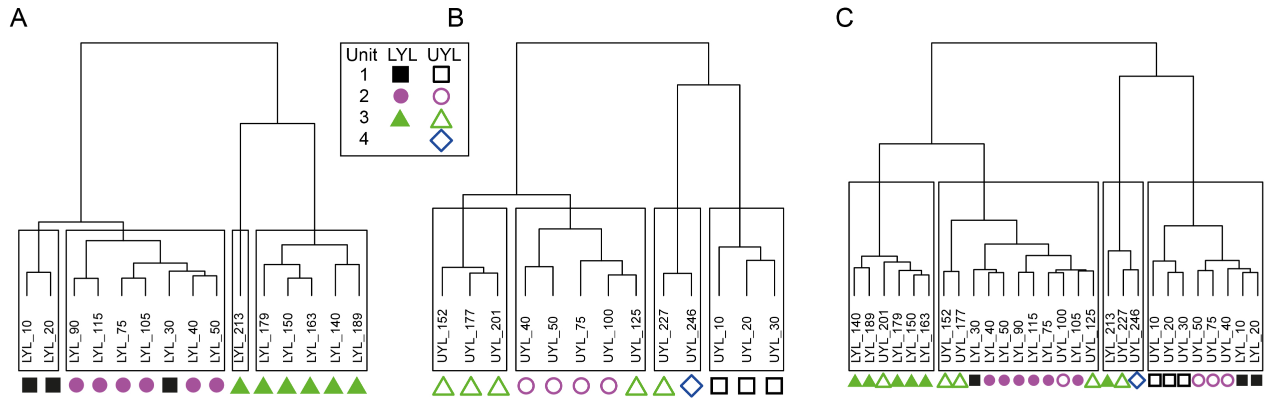

Figure 4: Cluster dendrograms for first and second axes of variance displayed in scatter plots of **(A-C)** PCA on the microbial community structure on class level, and **(D-F)** RDA of the microbial community composition in LYL on class level. Unit 1: black squares; unit 2: purple circles; unit 3: green triangles; unit 4: blue diamonds.

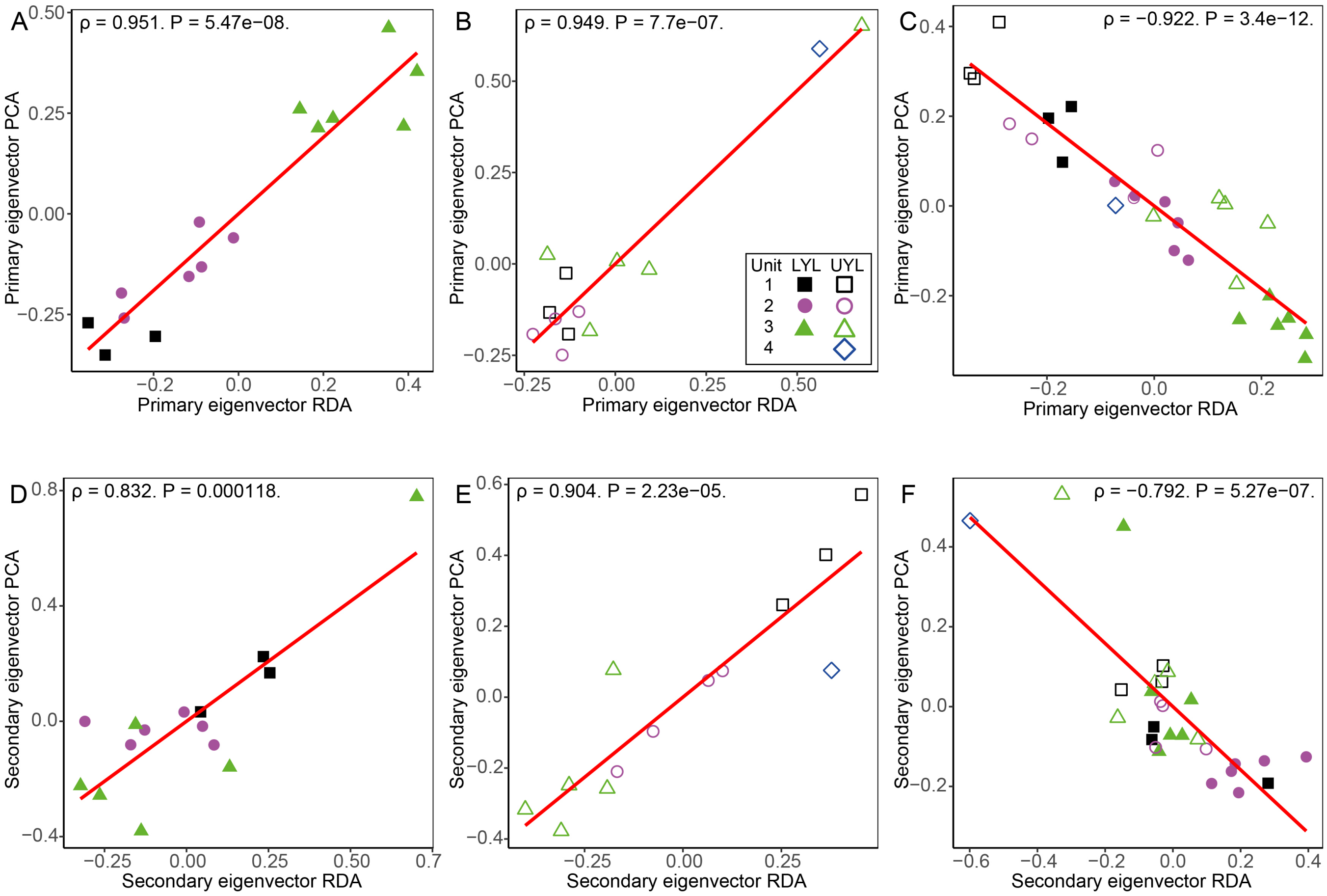

Figure 5: Correlations between the **(A-C)** primary eigenvector resulting from RDA of the microbial community structure on class level for LYL (filled shapes), UYL (hollow shapes), and YL constrained by the minimum adequate model, and the corresponding primary eigenvector resulting from PCA on the microbial community structure on class level. **(D-F)** Secondary eigenvectors. Unit 1: black squares; unit 2: purple circles; unit 3: green triangles; unit 4: blue diamonds.

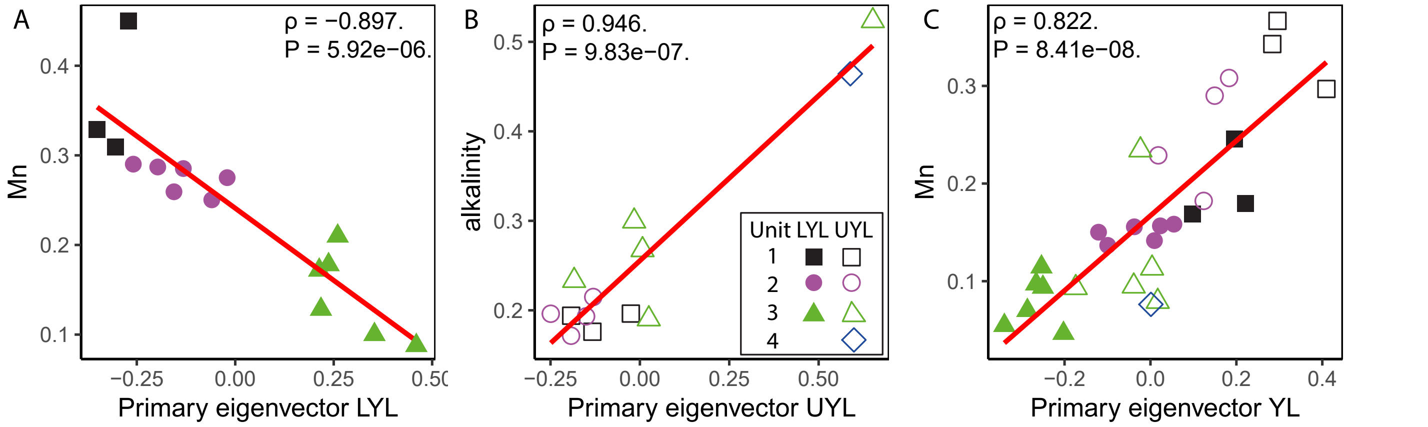

Figure 6: Correlations between the primary eigenvector resulting from PCA of the microbial community structure on class level, and the normalised context variable first selected as part of the minimum adequate model. **(A)** LYL (filled shapes), **(B)** UYL (hollow shapes), and **(C)** YL. Unit 1: black squares; unit 2: purple circles; unit 3: green triangles; unit 4: blue diamonds.

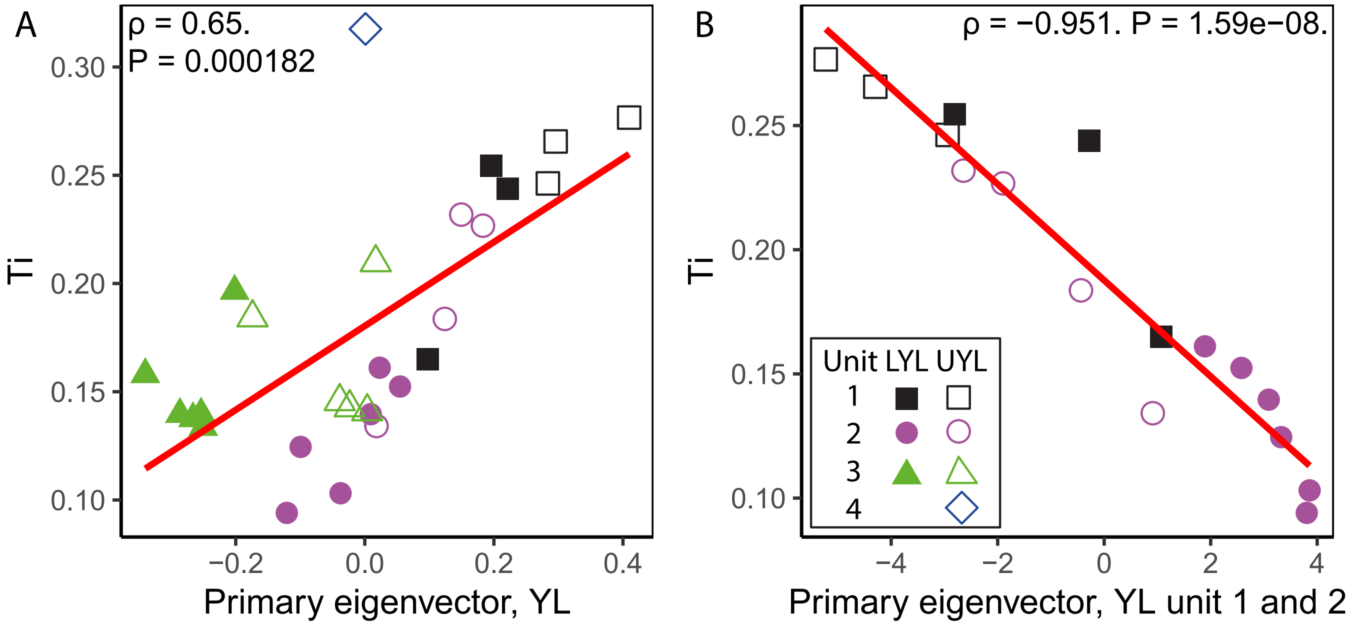

Figure 7: Correlations between the primary eigenvector resulting from PCA of the microbial community structure on class level, and normalised XRF counts of Ti. **(A)** YL, all units. **(B)** YL, unit 1 and 2. The eigenvector is based on PCA of unit 1 and 2 alone. Unit 1: black squares; unit 2: purple circles; unit 3: green triangles; unit 4: blue diamonds.

References

Cappelen, J., Laursen, E. V., Jørgensen, P. V., and Kern-Hansen, C. (2011). DMI Monthly Climate Data Collection 1768-2010, Denmark, The Faroe Islands and Greenland . Technical report, Danish Meteorological Institute.

Edgar, R. C. (2010). Search and clustering orders of magnitude faster than BLAST. *Bioinformatics*, 26(19):2460–2461.

Edgar, R. C. (2013). UPARSE: highly accurate OTU sequences from microbial amplicon reads. *Nature Methods*, 10:996–998.

Gloor, G. B., Macklaim, J. M., Pawlowsky-Glahn, V., and Egozcue, J. J. (2017). Microbiome Datasets Are Compositional: And This Is Not Optional. *Frontiers in Microbiology*, 8:2224.

Kolb, J. (2014). Structure of the Palaeoproterozoic Nagssugtoqidian Orogen, South-East Greenland: Model for the tectonic evolution. *Precambrian Research*, 255:809 – 822. Advances in understanding early Precambrian gneiss complexes.

Lanzén, A., Jørgensen, S. L., Huson, D. H., Gorfer, M., Grindhaug, S. H., Jonassen, I., Øvreås, L., and Urich, T. (2012). CREST – Classification Resources for Environmental Sequence Tags. *PLoS One*, 7(11):e49334.

Lecavalier, B. S., Milne, G. A., Simpson, M. J., Wake, L., Huybrechts, P., Tarasov, L., Kjeldsen, K. K., Funder, S., Long, A. J., Woodroffe, S., Dyke, A. S., and Larsen, N. K. (2014). A model of Greenland ice sheet deglaciation constrained by observations of relative sea level and ice extent. *Quaternary Science Reviews*, 102:54 – 84.

McMurdie, P. J. and Holmes, S. (2014). Waste Not, Want Not: Why Rarefying Microbiome Data Is Inadmissible. *PLOS Computational Biology*, 10(4):1–12.

Oksanen, J., Blanchet, F. G., Friendly, M., Kindt, R., Legendre, P., McGlinn, D., Minchin, P. R., O'Hara, R. B., Simpson, G. L., Solymos, P., Stevens, M. H. H., Szoecs, E., and Wagner, E. (2018). *vegan: Community Ecology Package*. R package version 2.5-2.

Pruesse, E., Quast, C., Knittel, K., Fuchs, B. M., Ludwig, W., Peplies, J., and Glöckner, F. O. (2007). SILVA: a comprehensive online resource for quality checked and aligned ribosomal RNA sequence data compatible with ARB. *Nucleic Acids Research*, 35(21):7188–7196.

van der Bilt, W. G. M., Rea, B., Spagnolo, M., Roerdink, D. L., Jørgensen, S. L., and Bakke, J. (2018). Novel sedimentological fingerprints link shifting depositional processes to Holocene climate transitions in East Greenland. *Global and Planetary Change*, 164:52–64.
